# Detection of Electronic Devices Using FMCW Nonlinear Radar

**DOI:** 10.3390/s22166086

**Published:** 2022-08-15

**Authors:** Kyuho Cha, Sooyoung Oh, Hayoung Hong, Hongsoo Park, Sun K. Hong

**Affiliations:** School of Electronic Engineering, Soongsil University, Dongjak-gu, Seoul 06978, Korea

**Keywords:** nonlinear radar, harmonic radar, FMCW, nonlinear RCS, nonlinear junction detection

## Abstract

Nonlinear radars can be utilized to detect electronic devices, which are difficult to detect with conventional radars due to their small radar cross sections (RCS). Since the receiver in a nonlinear radar is designed to only receive harmonic or intermodulated echoes from electronic devices, it is able to separate electronic devices from non-electronic scatters (clutter) by rejecting their echoes at fundamental frequencies. This paper presents a harmonic-based nonlinear radar scheme utilizing frequency-modulated continuous-wave (FMCW) signals for the detection of various electronic devices at short range. Using a laboratory experiment setup for FMCW radar at S-band for Tx (C-band for Rx), measurements are carried out to detect electronic devices of various sizes. The results show that the detection of small electronic devices is possible with nonlinear FMCW radar when appropriate system parameters are selected. Furthermore, we also discuss the maximum detectable range estimation for electronic targets using the radar range equation for FMCW nonlinear radar.

## 1. Introduction

Radars have been extensively used for the remote detection of objects for military and commercial applications since their first appearance in the 1930s [1,2]. Traditional radars use the same transmit and receive frequencies; that is, they detect the presence, distance, and speed of targets by receiving the transmitted pulses that are reflected back from the targets. In the sense that they transmit and receive the same frequencies, traditional radars are based on “linear” detection. For traditional radars, difficulties arise when it comes to detecting targets with small radar cross sections (RCS) in the midst of clutter or other large objects. Targets of interest with small RCS may include small electronic devices, which are typically situated in an environment that is surrounded by other objects with larger RCS, rendering limitations in detecting them.

To overcome this limitation of linear radars, “nonlinear” detection was proposed in the 1970s [3]. Due to the nonlinearities of electronic components and circuit elements in electronic devices, it is possible to separate small electronic devices of interest from linear scatterers, even in the presence of strong clutter and other objects with large RCS. Unlike linear radars, nonlinear radars use different transmit and receive frequencies. That is, by tuning the receiver to only receive the nonlinear responses (harmonic or intermodulation) that are excited by the nonlinear interaction at the semiconductor junctions in electronic devices, nonlinear radars allow for the detection of electronic devices in the probed environment [4]. Since natural linear scatterer and responses from other non-electronic objects do not produce nonlinear responses, it is possible to eliminate the linear response and only receive the desired nonlinear response from electronic devices. This allows for the effective detection of small electronic targets that would otherwise be difficult to achieve with linear radars [5,6,7,8,9,10,11,12]. Nonlinear radars can be utilized in various applications. For military and security purposes, they can be used to detect concealed electronic devices [13,14]. They have also been applied for detecting vital signs for health monitoring [15,16,17] and insect tracking using harmonic tags [18,19,20,21].

Recent studies have presented nonlinear radar systems using various types of waveforms, i.e., pulsed RF, continuous wave (CW), and chirp and stepped frequency continuous wave (SFCW) [8,22,23,24] for the detection of various nonlinear targets at various ranges. Since the signal processing and detection methods depend on the type of waveform that the radar transmits, it is important for radar systems to select an appropriate transmit waveform for a given application. In the case of detecting targets at short range (up to several meters), it is appropriate to transmit CW waveforms with modulation. An FMCW waveform, which consists of periodically repeated linearly modulated chirp signals, is appropriate for obtaining target information at short range [25,26,27]. The signal processing of FMCW radar is based on analyzing the beat frequency [27,28], that is, the frequency difference that is generated by mixing the transmitted and received signals through an RF mixer. For this reason, FMCW waveforms can also be applied to nonlinear (harmonic) detection at short range, where the radar would receive the harmonic version of the original transmit signals, and the beat frequency is generated by mixing the received signal with the frequency-doubled transmit signal.

In this work, we demonstrate the concept of an FMCW-based nonlinear radar for the detection of various electronic targets. The proposed system is a second-harmonic-based FMCW radar that uses S-band for transmit and C-band for receive with the aim of detecting electronic devices at short range (up to several meters). Using S/C band for harmonic radar may be advantageous compared to the previously reported UHF/L-bands [29] in terms of a smaller system size due to shorter wavelengths. Furthermore, compared to millimeter wave bands [14], utilizing S/C bands would allow for higher output power which can increase the maximum detectable range. The organization of the paper is as follows. In Section 2, the nonlinear radar equation and nonlinear RCS of various electronic devices are considered. The range equation for nonlinear FMCW radar is then presented. Section 3 presents the experimental apparatus that was used for the FMCW nonlinear radar measurement. In Section 4, the measured results are presented and discussed, where it shows that the targets are properly detected with their range information. The estimation of maximum detectable range based on the derived nonlinear FMCW radar equation is also presented. Section 5 concludes the paper with a quick summary and implications of the results.

## 2. Nonlinear Radar Equation, Nonlinear RCS, and Nonlinear FMCW Radar

Nonlinear radars require a different set of analyses from the conventional linear radars since the nonlinear interaction and scattering must be considered in the process. Therefore, it is essential to analyze the radar equation for the nonlinear radar that accounts for the nonlinear interaction/scattering from the target. In this section, we consider the nonlinear radar equation and discuss the apparent and nonlinear RCS. Furthermore, the radar range equation that is specific to the FMCW nonlinear radar is derived.

### 2.1. Nonlinear Radar Equation

**Figure 1:**
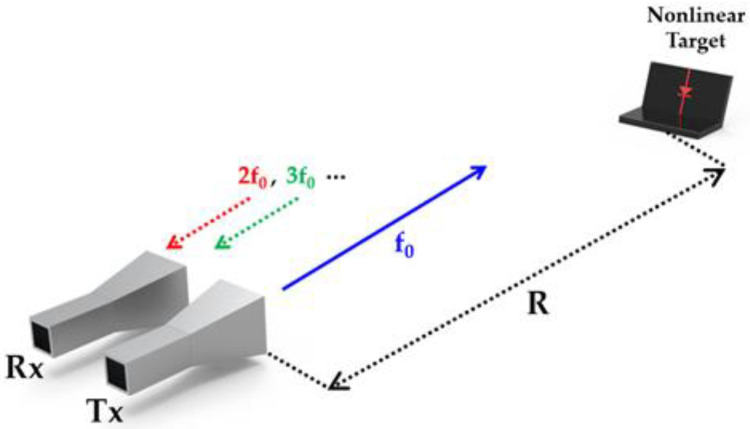
An illustration of a harmonic-based nonlinear detection.

Figure 1 illustrates nonlinear detection with a separate transmitter (Tx) and receiver (Rx) for an electronic target at a distance of R [m]. First, we start with the traditional linear radar equation format, i.e.,:

(1) $$P_{r}=\frac{P_{t}G_{t}G_{r}\lambda^{2}\sigma }{{\left(4\pi \right)}^{3}R^{4}}$$

where $\sigma \;[m^{2}]$ is the target RCS, $P_{t}\;[\mathrm{dBm}]$ is the transmit power and $G_{t}\;[\mathrm{dB}]$ is the gain of the transmitting antenna, $G_{r}$ is the gain of the receiving antenna, and λ [m] is the wavelength. For harmonic detection, the values of $G_{r}$ and λ should be those at the harmonic frequency. Equation (1) can be re-expressed by separating the transmit, scattering, and receive parts of the process as:

(2) $$P_{r}=\left[\frac{P_{t}G_{t}}{4\pi R^{2}}\right]\left[\sigma^{\prime }\right]\left[\frac{1}{4\pi R^{2}}\right]\left[G_{r}\frac{\lambda^{2}}{4\pi }\right]$$

The first term in Equation (2) describes the transmitted power density incident at the target. The second term is the “apparent RCS” which refers to the apparent size of the target that is perceived by the radar [30]. Note that σ is changed to $\sigma^{\prime }$ to indicate the apparent RCS. The third term represents the free space spreading loss. The last term describes the effective aperture of the Rx antenna. Hence, the total received power $P_{r}$ is the product of the abovementioned terms.

**Figure 2:**
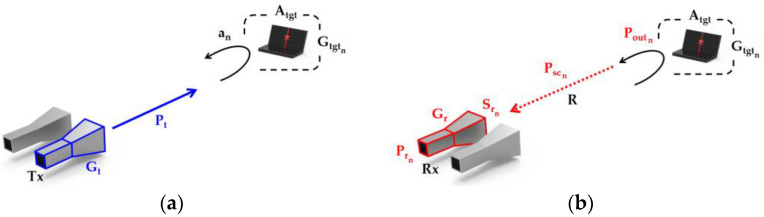
(**a**) Transmit and (**b**) receive process in nonlinear detection.

Figure 2 illustrates each process of nonlinear radar from transmit to receive. Here, we consider the nonlinear interaction at the target by treating the target as an effective antenna as suggested by Gallagher et al. [29,30]. By applying the nonlinear interaction at the target into Equation (2), derivation of the nonlinear radar equation is possible through a few additional steps. The transmitted power density incident at the target can be expressed as the first term of Equation (2). Assuming the target has an effective area of *A_tgt_* [m^2^], the power that is received by the target, *P_in_*, can be expressed by taking the product of the first term in Equation (2) and *A_tgt_* as:

(3) $$P_{in}=\frac{P_{t}G_{t}}{4\pi R^{2}}A_{tgt}$$

The power that is excited from the nonlinear interaction by the target, $P_{out}$, can be represented with a power series (assuming memoryless), i.e., [31]:

(4) $$P_{out}=\sum_{n=1}^{\infty }\left[a_{n}{\left(P_{in}\right)}^{n}\right]$$

where $a_{n}$ is a scaling coefficient of the $n^{th}$ harmonic that is produced by the nonlinear characteristics of the target. The power at the $n^{th}$ harmonic, $P_{out_{n}}$, can be extracted from the power series in Equation (4) and be expressed as:

(5) $$P_{out_{n}}=a_{n}{\left(P_{in}\right)}^{n}$$

Therefore, when the effective transmission gain of the target at the $n^{th}$ harmonic frequency is given as $G_{tgt_{n}}$, the scattered power from the target can be expressed as:

(6) $$P_{sc_{n}}=G_{tgt_{n}}\cdot P_{out_{n}}$$

As $P_{sc_{n}}$ travels back the distance of *R* towards the Rx antenna, the incident received power density, $S_{r_{n}}$, can be obtained as:

(7) $$S_{r_{n}}=\frac{P_{sc_{n}}}{4\pi R^{2}}$$

By rearranging the terms in Equation (7) using Equations (3)–(6) to be in line with Equation (2) by separating the transmit, scattering, and receive terms, we obtain:

(8) $$S_{r_{n}}=\left[\frac{P_{t}G_{t}}{4\pi R^{2}}\right]\left[G_{tgt_{n}}a_{n}A_{tgt}^{n}{\left(\frac{P_{t}G_{t}}{4\pi R^{2}}\right)}^{n-1}\right]\left[\frac{1}{4\pi R^{2}}\right]$$

The second term in Equation (8) is the apparent RCS $\sigma^{\prime }$ which corresponds to the $n^{th}$ harmonic response that is scattered from the target. The apparent RCS can then be expressed by separating the radar-specific terms and target-specific terms as [29]:

(9) $$\sigma^{\prime }=\frac{{\left(P_{t}G_{t}\right)}^{n-1}}{{\left(4\pi \right)}^{n-1}R^{2n-2}}\sigma_{n}$$

where $\sigma_{n}$ is defined as the nonlinear RCS of the target, which describes the physical and nonlinear characteristics of the target and can be expressed as [31]:

(10) $$\sigma_{n}=a_{n}G_{tgt_{n}}{\left(A_{tgt}\right)}^{n}$$

Since $\sigma^{\prime }$ is the apparent RCS as seen by the radar, it has the unit of [$m^{2}$]. But unlike linear RCS its value depends on the transmit power, gain, and distance, which determines the incident power level at the target as indicated in Equation (9). Depending on the incident power level at the target, the nonlinear response from the target varies. On the other hand, $\sigma_{n}$ describes the physical and nonlinear characteristics of the target at the *n^th^* harmonic, which is regarded as the true RCS of nonlinear targets at the corresponding harmonic frequency. Therefore, the unit of $\sigma_{n}$ depends on the harmonic order and is expressed as [$m^{2}/\left(W^{n-1}/m^{2n-2}\right)$].

The total received power $P_{r_{n}}$ can then be obtained by the product of $S_{r_{n}}$ and the effective area of the Rx antenna as:

(11) $$P_{r_{n}}=\left[\frac{P_{t}G_{t}}{4\pi R^{2}}\right]\left[G_{tgt_{n}}a_{n}A_{tgt}^{n}{\left(\frac{P_{t}G_{t}}{4\pi R^{2}}\right)}^{n-1}\right]\left[\frac{1}{4\pi R^{2}}\right]\left[G_{r}\frac{\lambda^{2}}{4\pi }\right]$$

By using Equations (9) and (10), Equation (11) can be expressed using $\sigma_{n}$ as:

(12) $$P_{r_{n}}=\frac{{\left(P_{t}G_{t}\right)}^{n}G_{r}\lambda^{2}\cdot \sigma_{n}}{{\left(4\pi \right)}^{n+2}R^{2n+2}}$$

which is referred to as the nonlinear radar equation [7,29]. The derivation above implies that one can obtain $\sigma_{n}$ of a nonlinear target at the corresponding harmonic from the experimental or calculated $\sigma^{\prime }$. Alternatively, Equation (12) can be directly used to obtain $\sigma_{n}$ from $P_{r_{n}}$.

**Figure 3:**
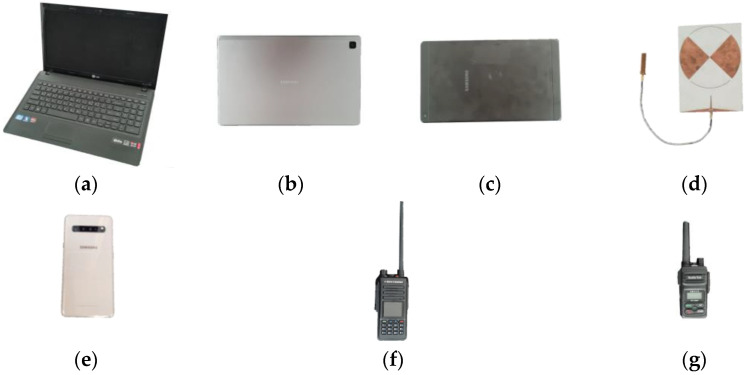
Various sizes of electronic targets that were used for measurement: (**a**) laptop, (**b**) tablet PC 2, (**c**) tablet PC 1, (**d**) diode-terminated antenna, (**e**) cell phone, (**f**) radio 2, and (**g**) radio 1.

In our recent work, we have conducted a measurement to obtain the apparent and nonlinear RCS of various electronic devices [30]. In the measurement, CW was used as the transmitted signal at 3.1 GHz of the fundamental frequency with 43 dBm of transmit power. Various types of electronic devices are selected as targets and located at 2.5 m from the transmit and receive antennas. Figure 3 shows the targets of interest that were used in the experiment, which are the targets that were used for the FMCW nonlinear radar experiment that are presented in this paper.

**Figure 4:**
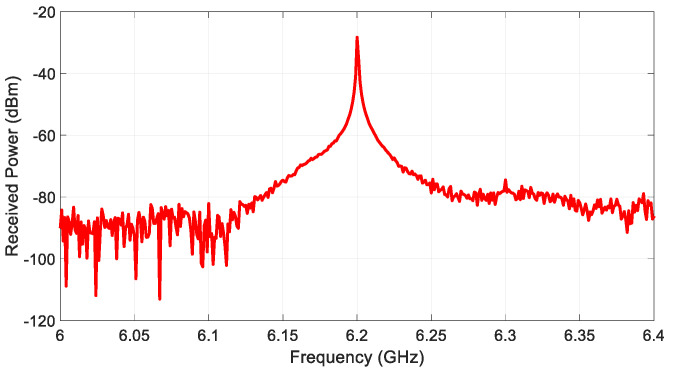
An example of the received harmonic response from target (laptop).

Figure 4 shows an example of the received power of harmonic response,$P_{r_{2}}$, from one of the targets (i.e., laptop). It can be seen that −32.04 dBm is received at the second harmonic frequency of 6.2 GHz. Table 1 summarizes the types and physical cross–sections of the selected nonlinear targets and the received power level of the harmonic responses of each target.

The second harmonic nonlinear RCS, $\sigma_{2}$, of each measured target that is listed in Table 1 is calculated using Equation (9) from the measured received power. It is demonstrated that the values of the calculated nonlinear RCS are within the general range of nonlinear RCS (second harmonic) of 10^−8^–10^−5^ [$m^{2}/\left(W/m^{2}\right)$] as suggested in previous studies [7,32]. Furthermore, it can be seen that the value of $\sigma_{2}$ and the received power is roughly proportional to the physical size of the target. Such a relationship can be attributed to two possible reasons: (1) the increase in the physical size of the target generally follows the size of the electronic boards within the target, which in turn increases $G_{tgt_{n}}$ and $A_{tgt}$ of the target, and (2) the increase in the electronic board size should also be roughly proportional to the number of semiconductor devices, which may result in a higher level of excited harmonic response (i.e., increase in the values of $a_{n}$).

**Figure 5:**
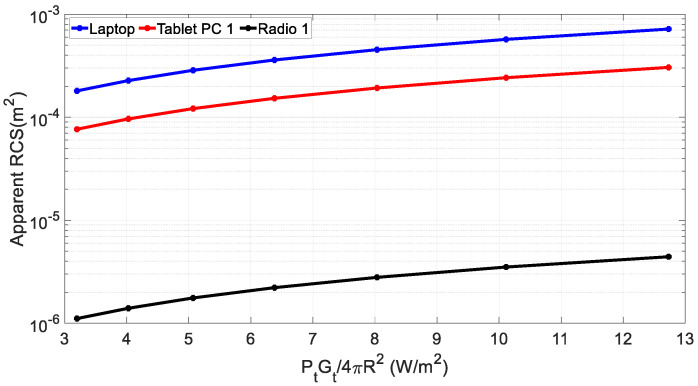
Measured apparent RCS of targets vs. the incident power density.

For a given target with $\sigma_{2}$, the apparent RCS can vary depending on the incident power level as indicated in Equation (9). To validate this, we obtain the values of $\sigma^{\prime }$ at different incident power density levels $\left({P_{t}G_{t}/4\pi R}^{2}\right)$ by varying $P_{t}$ for three targets with the largest, middle, and smallest $\sigma_{2}$. Figure 5 shows the values of $\sigma^{\prime }$ for these targets at a distance of 2.5 m at three different incident power density levels, where it can be seen that the value of $\sigma^{\prime }$ indeed increased for all the targets as $P_{t}$ increases.

### 2.2. Estimation of Maximum Detectable Range for FMCW Radar

The radar equation can be used to estimate the maximum detectable range. Here, to estimate the maximum detectable range for a second harmonic nonlinear FMCW radar, Equation (12) is modified in the following manner. First, changing $P_{r_{2}}$ into $S_{\min}$(the minimum receivable power) and equating with R, Equation (12) can be expressed as:

(13) $$R={\left(\frac{{\left(P_{t}G_{t}\right)}^{2}G_{r}{\lambda_{2}}^{2}\cdot \sigma_{2}}{{\left(4\pi \right)}^{4}S_{\min}}\right)}^{1/6}$$

$S_{\min}$ can be expressed as $kT_{e}BF{\left(SNR\right)}_{\min}$, where $kT_{e}B$ is the thermal noise in the system, with k being the Boltzmann constant, $T_{e}$ being the temperature, and B being the bandwidth of the receiver (second harmonic band) which needs to be the twice the transmit bandwidth. F is the noise figure of the receiver and ${\left(SNR\right)}_{\min}$ is the minimum signal to noise ratio that is allowed for detection. Applying the aforementioned terms, the maximum detectable range can be expressed as:

(14) $$R_{\max}={\left(\frac{{\left(P_{t}G_{t}\right)}^{2}G_{r}{\lambda_{2}}^{2}\sigma_{2}}{{\left(4\pi \right)}^{4}kT_{e}BF\cdot {\left(SNR\right)}_{\min}}\right)}^{1/6}$$

Next, Equation (14) is modified to account for the FMCW radar. To this end, frequency modulation bandwidth and the chirp duration need to be considered, which results in the processing gain (*PGR*) [33], i.e.,:

(15) $$PGR=BT_{chirp}$$

In general, loss exists throughout the radar system, which can be denoted by *L*. Considering *PGR* and *L*, Equation (14) can be re-written as:

(16) $$R_{\max}={\left(\frac{{\left(P_{t}G_{t}\right)}^{2}G_{r}{\lambda_{2}}^{2}\sigma_{2}}{{\left(4\pi \right)}^{4}kT_{e}BFL\cdot {\left(SNR\right)}_{\min}}PGR\right)}^{1/6}$$

which can also be expressed as:

(17) $${\left(SNR\right)}_{\min}=\frac{{\left(P_{t}G_{t}\right)}^{2}G_{r}{\lambda_{2}}^{2}\sigma_{2}T_{chirp}}{{\left(4\pi \right)}^{4}kT_{e}FL{R_{\max}}^{6}}$$

Based on Equation (16) or (17), the maximum detectable range or minimum *SNR* at a given range can be estimated for the detection of targets using harmonic FMCW radar.

## 3. FMCW Nonlinear Radar Measurement Setup

**Figure 6:**
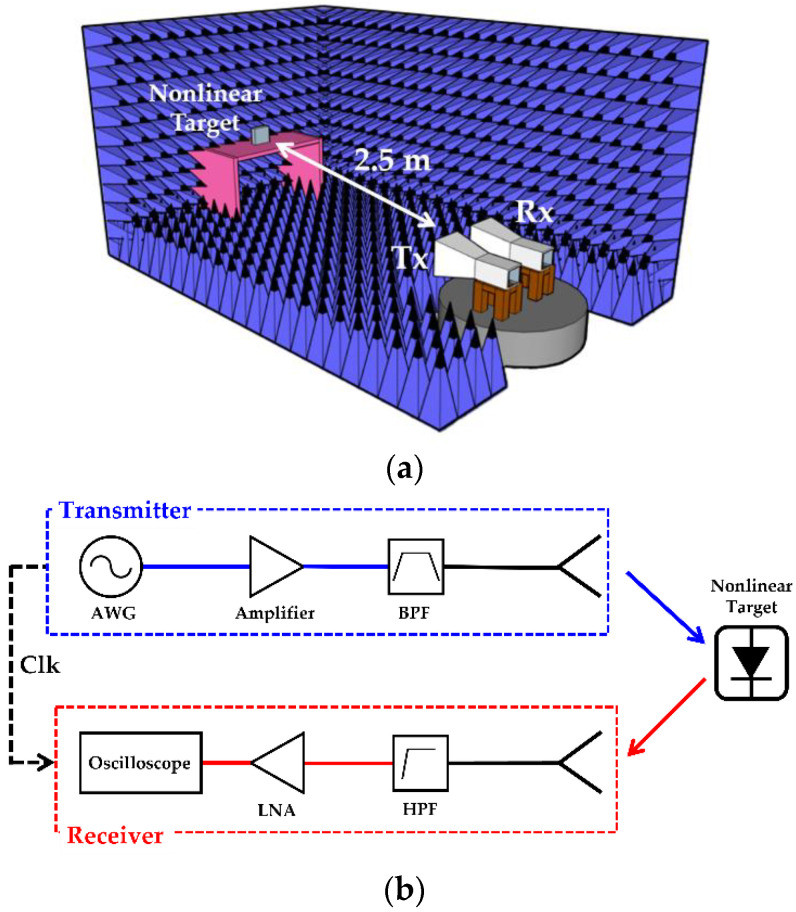
Nonlinear FMCW radar measurement setup: (**a**) Measurement environment in anechoic chamber and (**b**) block diagram of setup.

Figure 6a,b, respectively, show the measurement setup for nonlinear radar in an anechoic chamber and an overall block diagram of the measurement setup. Based on this block diagram, a measurement apparatus is established for detecting a target that was located 2.5 m away from the antennas. In nonlinear detection, the amplitude of the harmonic responses of targets is significantly lower than that of the fundamental responses. Thus, it is crucial to have sufficient transmit power and gain of Tx/Rx antenna. Moreover, the suppression of unwanted harmonics that are generated from the transmit side is also important to distinguish the desired harmonic responses from others.

**Figure 7:**
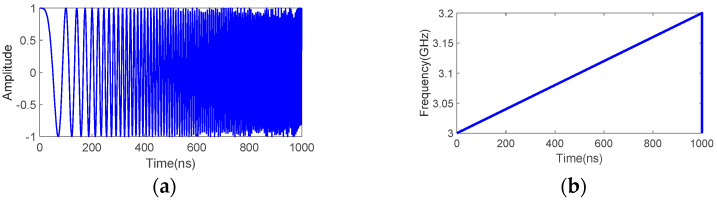

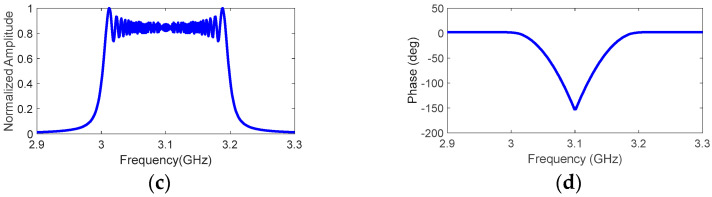
Transmitted FMCW signal that was used in the measurement: (**a**) time domain, (**b**) time-frequency domain, frequency domain (**c**) amplitude, and (**d**) phase.

The transmitter part consists of an AWG7102 arbitrary waveform generator to directly generate the transmit FMCW waveforms over a bandwidth of 3–3.2 GHz. The waveforms are then amplified using a 5183 RF power amplifier to provide a transmit power of 33 dBm (2 Watt) followed by a band pass filter before being transmitted through an S-band standard gain horn with a gain of 16.7 dBi. The transmit FMCW signal s(t) that was used in the measurement is generated based on the following equation:

(18) $$s\left(t\right)=A\cdot \cos\left\{2\pi \left(f_{start}+\mu t\right)t+\phi \right\},\;0\leq t\leq T_{s}$$

where $f_{start}$ is the start frequency set at 3 GHz and μ is the chirp rate defined as $\mu =B_{chirp}/2T_{S}$. Here, $B_{chirp}$ is set at 200 MHz and μ is set to have a chirp duration $T_{s}$ of 1 *μ s*. Figure 7 shows the transmit FMCW waveform that is generated from Equation (18) for one chirp duration. In the transmit mode, *s(t)* is repeatedly transmitted in a continuous manner.

On the receiving end, the operating bandwidth needs to be the second harmonic bandwidth of the transmit signals, i.e., 6–6.4 GHz. Therefore, a C-band standard gain horn antenna with a gain of 21 dBi is placed followed by two VHF-4600+ high pass filters that cover a bandwidth of 5.2–10.5 GHz for eliminating fundamental responses sufficiently and a ZX60-123SN-S+ low noise amplifier (LNA). A Tektronix TDS6154C fast-sampling oscilloscope is used to directly sample the received signals in the time domain. A clock signal that is generated from the AWG is directly fed into the oscilloscope to act as a reference signal. In order to minimize the unwanted harmonics from the source reaching the receiver, the Tx and Rx antennas are positioned with a slight offset. In a practical system, the received radar signals would pass through a mixer to be down-converted to baseband, where the signals contain the beat frequencies, which are then digitized for processing. However, note that in our experiment we perform the frequency down-conversion in the signal processing with directly sampled data.

## 4. Results and Discussion

### 4.1. Measured Second Harmonic Responses from Targets

**Figure 8:**
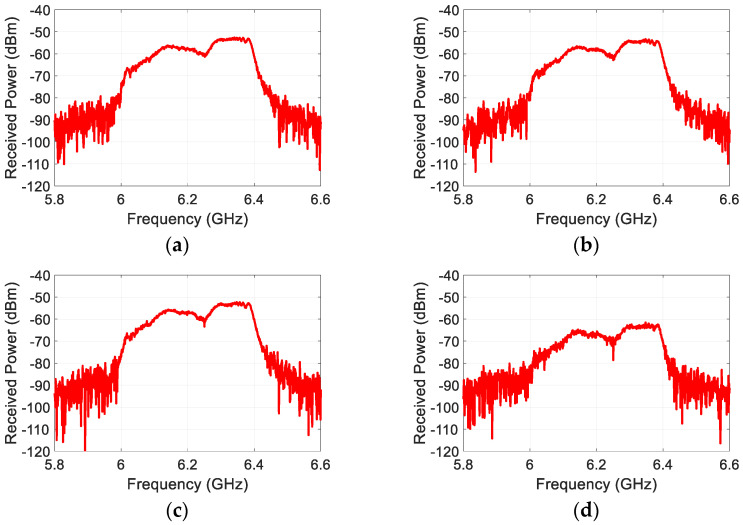

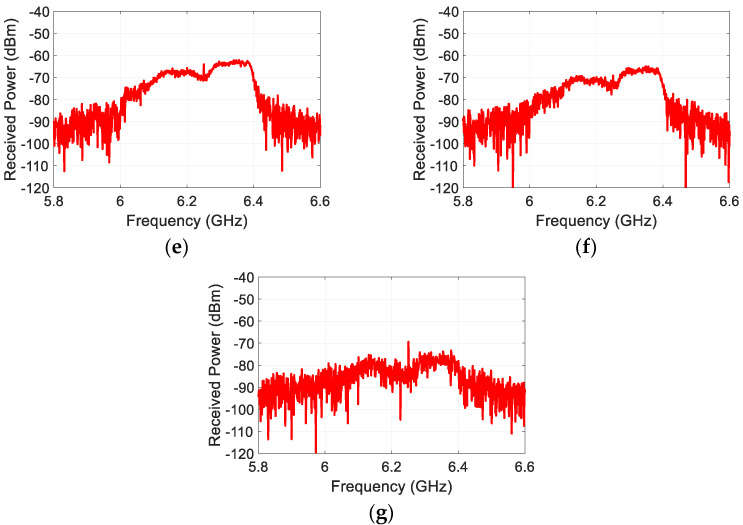
Amplitude spectrum of the measured target responses. (**a**) laptop, (**b**) tablet PC 2, (**c**) tablet PC 1, (**d**) cell phone, (**e**) diode-terminated antenna, (**f**) radio 2, (**g**) radio 1.

The second harmonic FMCW responses are measured for the electronic devices with various sizes as listed in Table 1. The time delay in the received harmonic responses includes not only the delay due to the target distance, but the delays that are caused by the system such as cable, filters, etc. Hence, it is essential for accurate target detection to separate unwanted time-delay from the total delay. In order to remove the system time-delay, a calibration measurement is performed before taking the target measurements. Figure 8 shows the spectrum of the received harmonic responses scattered from the targets that were measured. It can be seen that each target response is spread over 6–6.4 GHz, which is the second harmonic band of the transmit waveform. Also, the received power level of the target responses decreases in a similar manner to their nonlinear RCS in relation to their size, as expected. Note that the power levels that are shown in Figure 8 may seem lower since they include the total cable loss of about 12 dB in the measurement setup. The plots in Figure 8 are to show that detection of target responses was properly achieved in the measurement.

**Figure 9:**
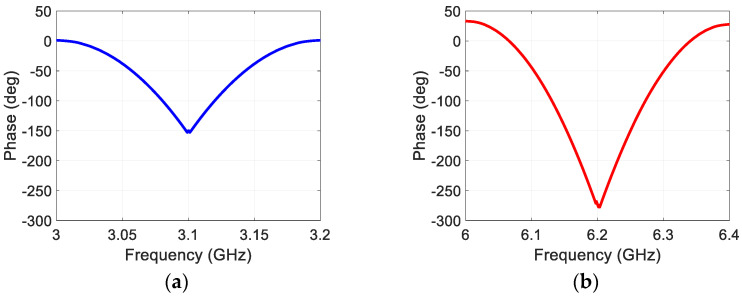
Phase plots of the (**a**) transmit and (**b**) receive (from the laptop) waveforms.

Since the target responses are the second harmonic version of the transmit FMCW waveform, their phase must exhibit a nonlinear characteristic as a function of the frequency. As an example, the phase of the response from the laptop in comparison to the transmit waveform is shown in Figure 9. In Figure 9a, the phase of the transmit waveform is shown, while in Figure 9b the phase of the harmonic response from the laptop is shown, over their respective frequency bands. It can be seen that the phase change in the harmonic response is twice that in the fundamental waveform as expected. Other target responses are also confirmed to exhibit the same phase behavior.

### 4.2. Extraction of Range Information

**Figure 10:**
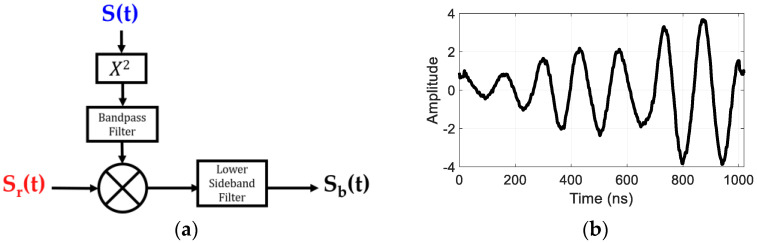
(**a**) Process of extracting the beat signal and (**b**) the extracted beat signal.

In the FMCW radar, the range information is obtained from the beat signal, $s_{b}(t)$, which is extracted by mixing the transmit and receive signals. The beat frequency occurs due to the time delay between the transmit and receive frequencies that is caused by the target distance. Here, we perform the mixing through signal processing by multiplying the transmit and receive signals and filtering out the lower band. For the second harmonic FMCW radar that was considered here, the frequencies of the transmit signal must be doubled to match the bandwidth with the received signals before mixing. Figure 10 illustrates the process of extracting the beat signal and shows an example of the extracted beat signal from a measured target response. After extracting the beat signals, the Fourier Transform of $s_{b}(t)$ is taken, which provides a compressed target echo, and the target distance information can be calculated by using the following relationship:

(19) $$R=\frac{cT_{s}}{2B}\times f_{b}$$

where c is the wave velocity, and $f_{b}$ is the baseband (beat) frequency.

**Figure 11:**
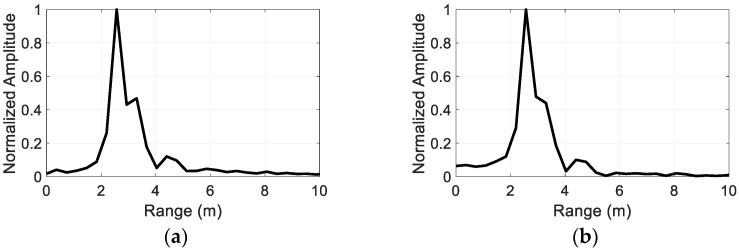

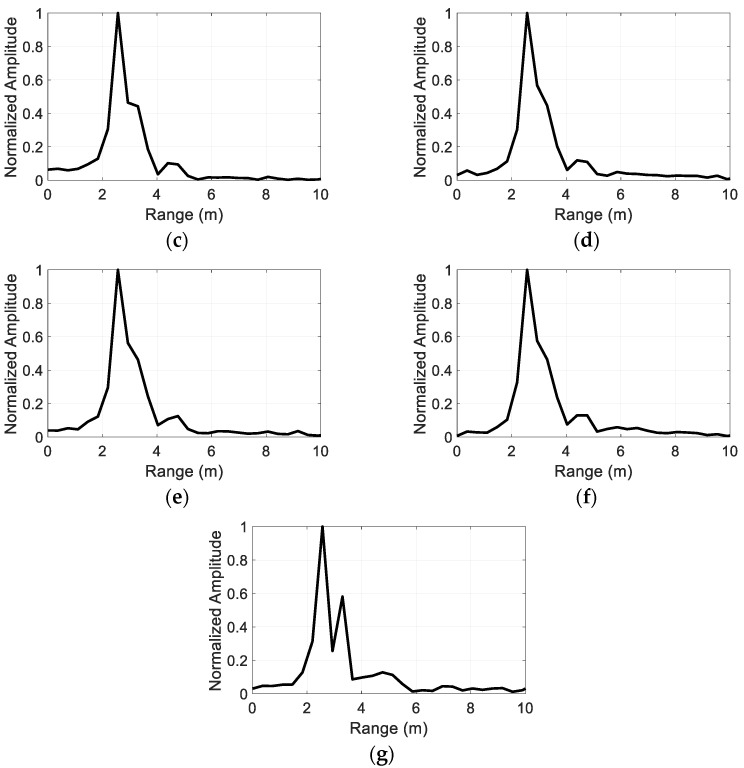
Compressed target echo as a function of the range for each measured target. (**a**) laptop, (**b**) tablet PC 2, (**c**) tablet PC 1, (**d**) diode-terminated antenna, (**e**) cell phone, (**f**) radio 2, (**g**) radio 1.

Figure 11 shows the compressed echo as a function of the range for the measured targets. Since the range is fixed at 2.5 m for all the targets, the peak of the Fourier Transform of the beat signals occurs at 6.82 MHz, which is approximated to be 2.57 m using Equation (19). The detected range seems to be in a good agreement with the actual target range of 2.5 m.

Range accuracy can be improved by considering the beat frequency error [27], which refers to the difference between the ideal beat frequency and the measured beat frequency. The maximum beat frequency error (Δ*f*) can expressed as:

(20) $$\Delta f=f_{s}/N_{s}$$

where $N_{s}$ is the number of data sampled over one period of beat signal and $f_{s}$ is the sample rate. Therefore, the range error (resolution) is expressed as

(21) $$\Delta R=\frac{cT_{s}\Delta f}{2B}=\frac{cT_{s}}{2B}\times \frac{f_{s}}{N_{s}}$$

As shown in Equations (20) and (21), it is a trade-off relationship between B, $T_{s}$, $f_{s}$, and $N_{s}$. Therefore, it is important to determine the appropriate parameters for a desired range resolution in a given system. The parameters that were used in our measurement give the beat frequency error of 1 MHz, which corresponds to the range resolution of 0.375 m.

### 4.3. Nonlinear Detection in the Presence of Linear Scatterer

**Figure 12:**
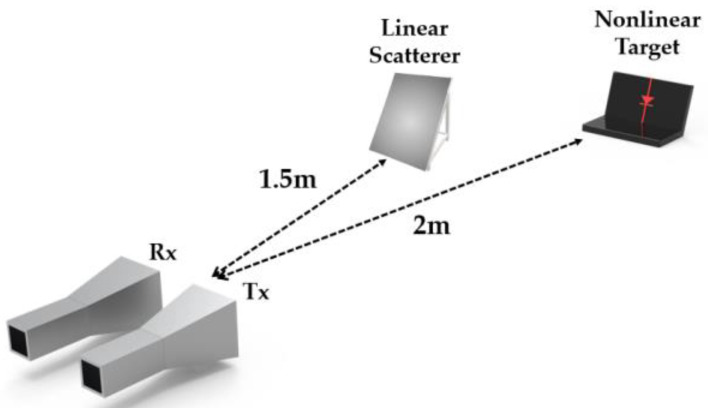
Nonlinear FMCW radar measurement setup with a linear scatterer.

**Figure 13:**
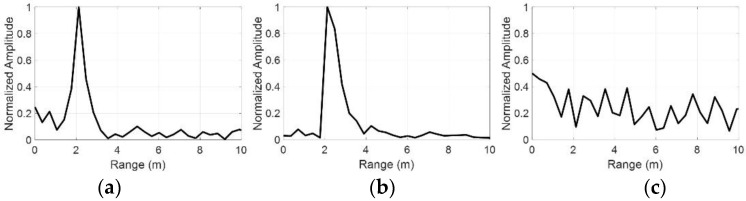
Compressed target echo as a function of the range: (**a**) laptop with linear scatterer (**b**) diode-terminated antenna with linear scatterer, and (**c**) only linear scatterers.

To test the proposed nonlinear radar’s ability to suppress linear scatter, the detection measurements were carried out with nonlinear targets in the presence of a linear scatterer. Figure 12 illustrates the measurement setup where the laptop and diode-terminated antenna are used as nonlinear targets and a rectangular metal plate (19 cm × 22 cm) as a linear scatterer. Here, the metal plate is located at a distance of 1.5 m from the antennas, and the nonlinear targets were placed at a distance of 2 m from the antennas. The received signals are processed in the same manner as described in Section 4.2, which can be seen in Figure 13. Figure 13a,b, respectively, show the compressed target echo of the laptop and diode-terminated antenna that were measured in the presence of the metal plate, as a function of the range. The peak of the Fourier Transform of the beat signals occurs at 5.66 MHz, which approximately corresponds to 2.12 m. The detected range seems to be in good agreement with the actual range of 2 m of the nonlinear targets. On the other hand, there is no peak that occurs around 1.5 m, indicating that the linear scatterer is not detected. Figure 13c shows the resulting compressed signal when the metal plate and antenna with an open circuit are present. Note that the antenna with the open circuit is the same antenna as the diode-terminated antenna, but with the diode removed, making it a linear scatterer. In this case, both targets are linear scatterers, resulting in no significant peaks at any of the distances, as expected. The results demonstrate that the proposed nonlinear radar can suppress responses from linear scatterers and detect the nonlinear target of interest in the midst of the linear scatterer.

### 4.4. Estimation of Maximum Detectable Range

**Figure 14:**
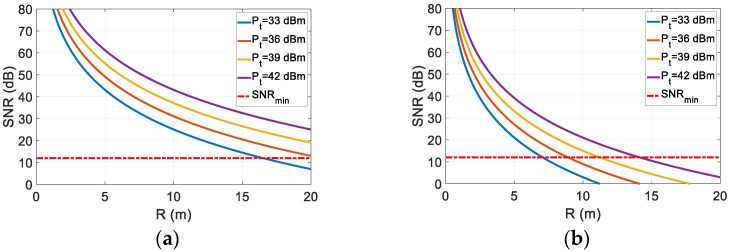
Estimation of the maximum detectable range for: (**a**) the laptop and (**b**) radio 1 based on the presented FMCW nonlinear radar parameters.

When designing a radar system, it is important to estimate the maximum detectable range and appropriate transmit power. For the harmonic FMCW radar that was considered here, Equation (17) can be used to estimate the maximum detectable range for a given target. Here, we estimate the maximum detectable range for some of the targets by using their experimentally obtained nonlinear RCS in Table 1. The thermal noise (*kT_e_B*) is estimated to be −87.9 dBm using the standard room temperature. An overall receiver loss (*L*) of 5 dB is assumed. The noise figure (*F*) is set to 2 dB. Figure 14 shows the estimated maximum detectable range for two representative targets (i.e., laptop, radio 1) for various transmit power levels (33–42 dBm). The minimum allowable signal to noise ratio ($SNR_{\min}$) is set at 12 dB as indicated by the red dashed line in the figure. It can be seen that the maximum detectable range for the laptop is approximately 16 m for a transmit power of 33 dBm. Furthermore, it is estimated that at least 39 dBm of transmit power is required to detect radio 1 at distances that are greater than 10 m. This estimation seems to support the measured detection results, where the smallest target (radio 1) is detected with a good level of signal to noise ratio at a 2.5 m range. However, the values of the nonlinear RCS can change according to each target’s physical characteristics and nonlinearities of the included electronic circuit. Therefore, this range estimation can be utilized when designing an FMCW nonlinear radar system while considering the aforementioned elements, thereby allowing for the selection of appropriate system parameters for detecting various electronic targets.

## 5. Conclusions

In this paper, we demonstrate a second harmonic-based FMCW nonlinear radar concept for the detection of electronic devices. With a laboratory experimental apparatus, FMCW nonlinear radar measurement is presented for various electronic devices. The results indicate the successful detection of small electronic devices with nonlinear FMCW radar when appropriate system parameters are selected. Here, the detection of only stationary targets is considered. Since FMCW radar is widely utilized for detecting moving targets, it is also possible to detect electronic devices in motion in the midst of linear scatterers with further processing. The proposed FMCW nonlinear radar can be utilized in applications such as security, military, and search and rescue, where obstructed or concealed electronic devices need to be detected in the presence of linear scatterer.

## Figures and Tables

**Table 1:** Various nonlinear targets and their estimated nonlinear RCS based on the received power [30].

Nonlinear Target (Size, ${\mathrm{cm}}^{2}$)	Received Power [dBm]	Nonlinear RCS [ $m^{2}/\left(W/m^{2}\right)$]
Radio 1 (5 × 9)	−54.15	3.4 × 10^−7^
Radio 2 (5.5 × 12)	−47.78	1.5 × 10^−6^
Diode-terminated Antenna (9 × 13)	−46.31	2.11 × 10^−6^
Cell Phone (6.7 × 18.8)	−42.99	4.53 × 10^−6^
Tablet PC 1 (12.4 × 21)	−35.76	2.39 × 10^−5^
Tablet PC 2 (15.7 × 24.7)	−32.61	4.94 × 10^−5^
Laptop (35 × 22)	−32.04	5.63 × 10^−5^

## Data Availability

Data sharing is no applicable.
